# Safety and Efficacy of Short Daily Hemodialysis with Physidia S^3^ System: Clinical Performance Assessment during the Training Period

**DOI:** 10.3390/jcm11082123

**Published:** 2022-04-11

**Authors:** Hafedh Fessi, Jean-Christophe Szelag, Cécile Courivaud, Philippe Nicoud, Didier Aguilera, Olivia Gilbert, Marion Morena, Michel Thomas, Bernard Canaud, Jean-Paul Cristol

**Affiliations:** 1 Nephrology Department, Tenon Hospital, 75020 Paris, France; hafedh.fessi@aphp.fr; 2 Dialysis Department, Aural Dialysis Center, 69008 Lyon, France; jean-christophe.szelag@artic42.fr (J.-C.S.); p-nicoud@ch-hopitauxduleman.fr (P.N.); 3 Nephrology Department, University Hospital Center of Besancon, 25030 Besancon, France; ccourivaud@chu-besancon.fr; 4 Nephrology Department, Léman Hospital, 74200 Thonon Les Bains, France; 5 Nephrology Department, Hospital Center of Vichy, 03200 Vichy, France; didier.aguilera@ch-vichy.fr; 6 Charles Mion Foundation, AIDER-Santé, 34090 Montpellier, France; o.gilbert@aidersante.com (O.G.); canaudbernard@gmail.com (B.C.); 7 PhyMedExp, University of Montpellier, INSERM, CNRS, Department of Biochemistry and Hormonology, University Hospital Center of Montpellier, 34000 Montpellier, France; m-morenacarrere@chu-montpellier.fr; 8 Physidia, 49124 Saint-Barthélemy-d’Anjou, France; michel.thomas@physidia.fr; 9 Emeritus Professor of Nephrology, University of Montpellier, 34000 Montpellier, France

**Keywords:** end-stage kidney disease, hemodialysis, home therapy, portable artificial kidney

## Abstract

Background: A growing body of scientific evidence indicates that clinical outcomes of hemodialysis patients can be improved with short daily dialysis treatment. Current in-center hemodialysis machines do not fulfill the requirements needed for self-care home hemodialysis (HHD) treatment. In line with the reviviscence of home therapy, several hemodialysis devices have been developed and deployed for treatment. Physidia S^3^ is one of these new dialysis delivery systems featuring an appealing design and functionalities intended for daily HHD treatment. Methods: In this French multicenter proof-of-concept study enrolling 13 training centers, we report our preliminary experience with a special focus on quantifying clinical performances in short daily HHD treatment performed during the training period of the patients. Results: Among the 80 patients included in this study, a total of 249 sessions could be analyzed. Dialysis dose, estimated from weekly standardized Kt/V, was maintained at 2.22 [1.95–2.61] with a normalized protein catabolic rate of 0.93 [0.73–1.18] g/kg/24 h. Furthermore, anemia and nutritional status were adequately controlled as indicated by 11.6 ± 1.4 g/dL of hemoglobin level and 39.4 ± 5.7 g/L of serum albumin as well as electrolyte disorders. Conclusions: The safety and efficacy of the S^3^ therapy concept relying on a short daily hemodialysis treatment using a bagged delivery system are in total agreement with daily HHD recommendations. Clinical performances are aligned to the metabolic needs of the vast majority of HHD patients. Currently ongoing studies at home will provide further evidence and value of this therapeutic approach.

## 1. Introduction

Accumulative scientific evidence indicates that clinical outcomes of hemodialysis (HD) patients can be improved with more frequent and/or longer treatment duration than conventional thrice-weekly HD [1,2,3,4]. Short daily HD results in smaller biologic fluctuations, better control of uremia, and lower ultrafiltration rate resulting in better patient perception [5,6]. Long-term studies also indicate that patients treated with daily HD have better intermediary outcomes that include lower blood pressure, better fluid status control, reduction of left ventricular mass, better nutritional status, and improved serum phosphorus levels [7,8]. Considering that short daily HD is mainly developed at home for practical reasons, this combination is likely to improve quality of life and reduce treatment costs [9,10]. 

Despite the attractiveness and many advantages of home HD (HHD), this treatment option remains underutilized worldwide with a low acceptance rate (below 0.5% in the US [11] and around 1–1.2% in France [12,13]) mainly driven by a short daily hemodialysis treatment schedule but interestingly with a significant growing share. Among the various barriers to the widespread use of short daily HHD over the past years was the lack of specifically designed HHD devices featuring small, lightweight devices and patient-friendly interfaces [14,15]. Basically, HHD devices should mitigate inherent risks associated with extracorporeal treatment and permit easy handling, time-saving set-up and reduced storing, in order to minimize burden for the patient and care partner [15,16]. 

Following renewing clinical interest for HHD, several companies and/or manufacturers have taken on this challenge and developed appealing HHD devices with various technical features (i.e., NxStage, QuantaSC+, Tablo) [17]. In this perspective, the Physidia S^3^ (S^3^) dialysis device was created with three main aims: firstly, to enable easy-going HHD treatment; secondly, to ensure safety and to optimize the efficacy of short daily HD; thirdly, to facilitate the integration of HD into the daily life of the patients and finally to significantly increase their quality of life. With these premises in mind, the S^3^ therapy concept was elaborated, relying on a short daily low-dialysate-flow delivering system from sterile bags and featuring with friendly and smart patient interface supported by a fully connected technology. Although the S^3^ device has been clinically used in France since 2013, little performance data have been published so far. Preliminary results mentioned a weekly urea Kt/V of 2.56 in a small population of 10 patients [18], and more recently, the use of this device with patients hospitalized for Covid-19 was reported [19].

The objectives of this retrospective study were to assess the weekly performance and safety of the device in a multicenter proof-of-concept study involving a large number of patients included during their training period.

## 2. Materials and Methods

### 2.1. Physidia S^3^ Description and Characteristics

**Figure 1:**
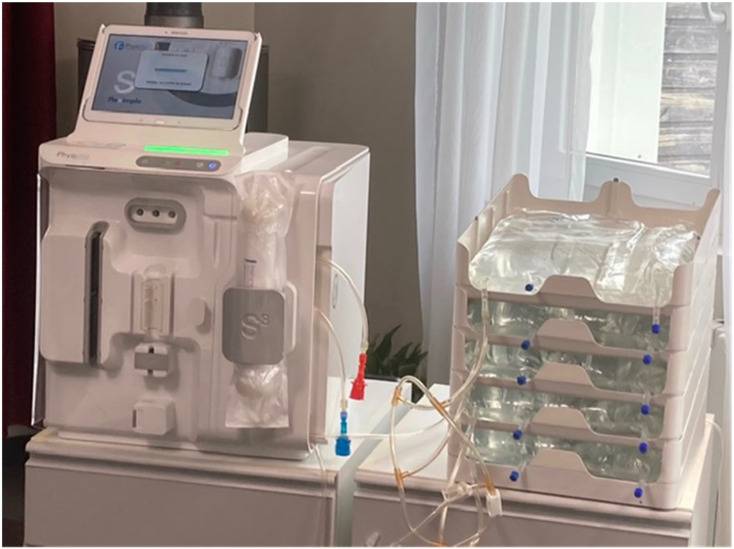
A Physidia S^3^ hemodialysis device.

The S^3^ device was developed in France (Physidia, Saint-Barthélemy-d’Anjou, France) from a medically proven concept relying on a bagged delivery and dialysis monitoring device [20]. This device has a compact and portable cubic design (dimension 40 × 40 × 40 cm) weighing less than 25 kilograms (Figure 1) and specifically designed for short daily low-dialysate-flow HHD. It integrates all technical and safety features for monitoring flow (blood, dialysate, ultrafiltrate) and pressure (blood, transmembrane pressure). The extracorporeal blood circuit relies on a dual needle system requiring the successful insertion of two needles (or dual venous catheter) for each dialysis treatment. The dialyzer not being pre-attached to the tubing set, the device allows a dialyzer personalized prescription by the physician. All dialysate is made in sterile bags (5 L) stored in a rack (up to 7 bags), warmed and delivered to the patient, and spent dialysate is discharged directly into the waste streams. The blood flow pump operates from 100 to 350 mL/min and dialysate flow is set from 150 to 200 mL/min. The balance chamber technology with adjustable ultrafiltration rate is able to provide substantial convective volume-enhancing internal filtration relying on a “push-pull” technology also called SeCoHD for “self-convective hemodialysis” which allows a better removal and a limited membrane fouling [21]. In addition, S^3^ has a cartridge set-up, automated prime function, removable tablet with a touch-screen patient interface, and the ability to store and transmit treatment data to the clinic patient file after treatment. It has an integrated automated blood pressure monitor but no integrated heparin pump. S^3^ device has been registered as a medical device and approved for in-center and HHD use with CE (European Conformity) marked in 2013.

### 2.2. Study Design

In this French multicenter retrospective study (post-market survey), clinical performances of S^3^ used in a short daily treatment schedule 5–6 days/week (intensified dialysis schedule) were analyzed throughout a 48-month period corresponding to the duration of the period of inclusion and of the training as a proof-of-concept study. Informed consent was obtained after acceptance of the HHD principle and before the training period. Thirteen training dialysis facilities collaborated on this assessment and provided patient data according to the number of dialysis patients enrolled. A common protocol of dialysis training was shared by all centers. Data collection, based on local practices, was performed under the supervision of a referent nephrologist and training caregivers during the training period preceding HHD installation. Data extraction was performed at the training facility level using the patient electronic medical records. Existing data were anonymized, sent, and pooled at the coordinator center level for further analysis. End-points for performances were weekly urea Kt/V, urea mass removal, phosphate reduction, and mass removal. Safety was evaluated by the report of complaints related to adverse events linked to the device.

### 2.3. Ethics Approval and Consent to Participate

All patients gave their informed consent to be enrolled in a home training program. The study was performed in accordance with the Good Clinical Research Practice guidelines, conducted in accordance with the declaration of Helsinki, and performed on the existing data registry after being anonymized, pooled, and analyzed. Blood sampling was part of regular patient monitoring during the training phase, and no extra blood sampling was taken for this study.

The study was approved by the Institutional Review Board committee of Montpellier University Hospital (IRB-MTP_2021_02_202100722).

### 2.4. Patients

Eighty chronic kidney disease stage 5 dialysis (CKD5D) patients voluntary for HHD program were enrolled and trained for HHD treatment. Upon time of treatment modality choice, patients were informed about HHD therapy and opted for daily treatment. All patients participated on purpose in a training program relying on a short daily HD treatment delivered with S^3^ and mimicking HHD therapy. The population analyzed consisted of 12 patients (incident) with end-stage kidney disease transitioning to dialysis and 68 stable dialysis patients (prevalent) who were switched to home treatment. The only exclusion criterion was age below 18 years old.

### 2.5. Hemodialysis Prescription

At baseline, standard HD prescription consisted of 5–6 treatments/week lasting 120 min, flowing dialysate at 180 ml/min (5 bags of 5 L) up to a 1- or 2-week testing period. After this probing period, the treatment prescription was then adjusted to patient needs and tolerance. The basic volume of fluid delivered was set at 20 L/session and could be adjusted up to 30 L/session according to dialysis adequacy results.

High-flux hemodialyzers were used in all cases. Dialyzer brand and surface area were left to the discretion of the referent physician. Proprietary single-use disposable tubing lines consisting of a blood tubing set (arterial and venous line with pressure sensor) and a dialysate tubing set (cassette) were used in all dialysis sessions across the entire population.

Dialysis treatment tolerance and efficacy were assessed on standard clinical and biological criteria. Patient monitoring consisted in capturing clinical symptomatology and measuring vital signs (i.e., weight gain/loss; blood pressure; heart rate; intradialytic events; temperature) during dialysis sessions. Treatment efficacy was assessed on clinical performances using selected parameters (i.e., fluid status, blood pressure control, standard weekly Kt/V, percent reduction of selected biomarkers, electrolyte and bone metabolism biomarkers) and tailored to results and patient needs relying on 5–6 days a week treatment schedule.

Training period time, including cannulation, self-care handling, and S^3^ machine learning, varied from patient to patient. 

### 2.6. Calculations

See Appendix A for Details.

- Dialysis dose.

The dialysis dose delivered was calculated using various indexes [6,22,23,24].

b. Solute mass removal.

Solute mass removal for solutes of interest (urea, creatinine, and phosphates) was calculated as previously described [25,26].

c. Percent reduction (PR) of solutes.

Percent reduction (PR) of solutes was calculated as follows:

$$\mathrm{PR}\;=\left[1-\left(\mathrm{Cpost}/\mathrm{Cpre}\right)\right]\times 100$$

where C is solute concentration; pre and post correspond to pre- and post-treatment conditions, respectively.

d. Normalized Protein Catabolic Rate (nPCR).

nPCR derived from urea apparition rate was calculated as previously described [27].

### 2.7. Statistical Analysis

Data analyses are only descriptive in this study. Qualitative variables are described by their proportion (percentage). Quantitative variables are expressed as mean values ± standard deviation (SD) for normally distributed variables and median (percentile 25–percentile 75) for non-normally distributed variables and calculated on the totality of data collected per patient and for the cohort. Distributions were tested using the D’Agostino and Pearson test.

Statistical analysis was performed using JASP 0.16.1 (JASP software, Amsterdam, The Netherlands) and GraphPad Prism 8.0.1 (GraphPad Software, San Diego, CA, USA).

## 3. Results

### 3.1. Patients’ Characteristics

Patients’ characteristics at baseline are reported in Table 1.

Primary cause of kidney disease was as follows [28]: primary glomerulonephritis (*n* = 14; 17.5%); cystic–hereditary–congenital (*n* = 12; 15.0%); hypertension–vascular disease (*n* = 9; 11.3%); diabetes (*n* = 3; 3.8%); neoplasm-tumor (*n* = 8; 10.0%); secondary glomerulonephritis-vasculitis (*n* = 12; 15.0%); interstitial nephropathy-pyelonephritis (*n* = 4; 5.0%); miscellaneous condition (*n* = 10; 12.5%); unknown (*n* = 8; 10.0%). 

Diabetes mellitus was present in 7.5% of patients. Arteriovenous fistula or graft (but no central venous catheter) was used in this study. 

Occupational distribution was classified as follows [29]: professionally active (83.8%), retired or pensioned (6.3%), disabled (8.8%), unemployed (0.0%) and unclassified (1.3%).

### 3.2. Operating Conditions—Treatment Characteristics

Median effective operating dialysis conditions were treatment time per session 120 (120–146) min, blood flow 280 (280–300) mL/min, dialysate flow 180 (180–180) mL/min, and enhanced convective volume induced by the adjustable “push-pull” technology 3.0 (2.0–3.0) L. The maximum blood flow rate was set at 300 mL/min, whatever the vascular access was to fit with the low dialysate flow rate.

High-flux hemodialyzers with a surface area between 1.5 and 2.2 m^2^ were used in all cases. The median dialysate volume spent per session (dialysis phase) was 22 (20–30) L, corresponding to an average cumulative weekly dialysate spent of 120 L/week. 

Electrolyte dialysate concentrations were as follows: Na^+^ 140 mmol/L, K^+^ 1 (91.3% of patients), 1.5 (6.3% of patients) or 2 (2.5% of patients) mmol/L, Ca^2+^ 1.5 mmol/L, Mg^2+^ 0.5 mmol/L, HCO_3_^−^ 35 mmol/L (95.1% of patients) or lactate 40 mmol/L (1.3% of patients) depending on medical patient profile, glucose 5.55 mmol/L. 

Anticoagulation of the extracorporeal circuit was required for 78.8% of patients and ensured by means of a single dose of low molecular weight heparin administered within the outlet tubing line at dialysis initiation. The remaining percentage of patients did not use anticoagulation due to the relatively short treatment time.

### 3.3. Treatment Sessions Analyzed

From the 80 patients included in this study, 249 sessions could be analyzed with a median value of 2 (1–4) sessions per patient. Nineteen patients carried out 5 or more sessions. All dialysis sessions were performed as prescribed and conducted till the end.

### 3.4. Safety and Difficulties Related to the Procedure during the Training Period

The training period varied from patient to patient, with a median training time per patient of 41 (28–56) days. Neither safety concerns related to the use of S^3^ (complaints related to adverse events linked to the device) nor side effects/difficulties related to the procedure have been observed among the patients during this period. In addition, none of the patients or families complained of the mass and volume of bags needed for each treatment. 

### 3.5. Clinical and Biological Performances

Fluid status and blood pressure control were easily achieved with this daily hemodialysis schedule, facilitated by a low ultrafiltration rate (8.5 (4.0–12.1) mL/h/kg). Ultrafiltration volume and body weight changes are presented in Table 2, biological parameters in Table 3, and clinical performances in Table 4. Equilibrated urea Kt/V per session was 0.59 (0.49–0.75), while expressed as weekly standardized Kt/V was 2.22 (1.95–2.61).

Percent reduction of selected biomarkers were 44.3 (38.7–52.9), 46.0 (40.0–54.5), and 44.4 ± 16.7% for urea, creatinine, and phosphate, respectively. Furthermore, an estimate of urea mass removed was 383 (283–515) mmol/session. 

### 3.6. Patient Perception and Acceptance Rate

After the training period, 78 patients (97.5%) were successfully installed at home and used the device for an average duration of 3.4 years (1255 (1561–3005) days). One patient failed in this training program due to poor compliance. One patient did not complete his home training due to kidney transplantation after 10 days of education. Ease of use and handling of S^3^ device was recognized by all patients and educated nursing staff. This holds true for fitting the dialyzer and tubing set, as well as loading dialysate bags and setting the S^3^ dialysis monitor via the tablet interface. The total handling time to set the S^3^ monitor and launch dialysis session was about 25 (20–35) minutes, including bag installation, rinsing, and priming phases.

## 4. Discussion

In this French multicenter study, we report clinical experience with Physidia S^3^ system specifically designed for daily HHD treatment. Thirteen training centers experienced in HHD were enrolled to evaluate clinical performances in treating chronic ESKD patients with S^3^. During this training period, 80 dialysis patients who accepted to be part of the HHD training program were treated and trained with this device in order to benefit from a short daily treatment program. 

The S^3^ dialysis device has specific features intended to facilitate self-therapy and HHD. S^3^ consists of a monitoring device composed of a central processing unit integrating information and algorithms capable of driving extracorporeal blood and dialysate circuits. S^3^ relies on a bagged dialysate delivery system that uses disposable sterile and non-pyrogenic dialysis fluid presented in 5-L bags stored and disposed of horizontally during dialysis session in a rack system. Dialysis fluid is pumped through the hemodialyzer at low flow, single-pass mode in a closed circuit while spent dialysate is wasted to drain. The specific dialysate circuit facilitates ultrafiltration control but also offers an additional option for increasing internal filtration (push-pull technic). This technical feature is intended to enhance the convective transport phenomenon and can increase the clearance of middle and larger molecular weight substances. Total convective volume obtained from both ultrafiltration as weight loss and forced internal filtration transport (“push-pull” technology) could achieve 4.3 L/session (1.3 weight loss and 3.0 push-pull system), corresponding to 26 L/week, which is in agreement with high-flux HD in ultrafiltration-controlled mode [21,30,31,32]. Of note, the convective volume given by the machine corresponds to the total volume ultrafiltered (without the weight loss), including the additional ultrafiltration performed via the push-pull-like technique. It does not take into account the “internal filtration” phenomenon (ultrafiltration/backfiltration) due to the pressure equilibrium in the capillary dialyzer imposed by the volumetric control of the S^3^ machine. In addition, 100% of incident patients with recent treatment initiation had a residual kidney function with a diuresis >0.5 L/day, and HHD was used as part of an incremental dialysis program [33,34,35]. The blood and dialysate circuits are independent disposable tubing sets presented in a sterilized package ready to use and easily inserted on the S^3^ device by the patient himself. S^3^ has a tactile tablet that offers a friendly and quite intuitive interface for guiding or helping patients in their care task. Of note, the training period for patients (of 41 (28–56) days) mainly included the self-puncture training. Only a 3 week-period is needed for the handling and training use of the S^3^ monitor.

As highlighted, S^3^ is fully designed and featured to facilitate self-therapy and HHD treatment relying on a dialysate delivery system and monitoring device excluding cumbersome tasks (i.e., water treatment system, disinfection) and/or burden of HD care management with a wireless connectivity tablet device (i.e., easy handling, portable device).

The patient training period was used to quantify clinical performances and to adjust weekly dialysis volume prescription of a short daily treatment program mimicking future home treatment conditions using S^3^. The median training period lasted 41 (28–56) days. Treatment schedule during this period consisted of five or six weekly dialysis sessions lasting 2 h (e.g., 12 h/week). During this period, 249 test dialysis sessions were used to assess and quantify dialysis session efficacy on usual parameters. 

Blood and dialysate flows were in agreement with prescription. Ultrafiltration rate and volume were concordant with patient weight loss and remained in close agreement with prescription. 

Clinical performances delivered with S^3^ assessed on usual dialysis adequacy markers confirmed the efficacy of the short daily hemodialysis treatment concept. In the majority of patients, treatment adequacy was achieved with regular use of 20 L/session of dialysis fluid delivered (e.g., 120 L/week). A limited number of patients required 3 h/day and the delivery of 30 L/session of fluid (e.g., 180 L/week) to achieve dialysis adequacy targets. 

Fluid volume control relying on dry weight achievement was achieved adequately by means of ultrafiltration volume and sodium removal in the majority of patients. As illustrated here, the daily treatment program was associated with a relatively low ultrafiltration rate (8.5 mL/h/kg), a condition that may preserve volemia, facilitate fluid volume control, and improve hemodynamic stability [36]. 

Solute removal performance and uremic control assessed on usual biomarkers indicated that short daily dialysis treatment program was able to achieve treatment adequacy. As reported, the median dialysis dose delivered, summarized by weekly standardized Kt/V, was 2.22 (1.95–2.61) and was associated with a median serum urea time average concentration of 14.2 (11.4–18.7) mmol/L while dietary protein intake estimated from nPCR was 0.93 (0.73–1.18) g/kg/24 h. This weekly level of dialysis dose delivered is fully in line with KDOQI recommendations concerning frequent dialysis since a target standard Kt/V of 2.3 per week with a minimum delivered dose of 2.1 is recommended for HD schedules other than thrice weekly [37], while 1.7 per week is recommended for peritoneal dialysis [38]. The very recent core curriculum for HHD established that the expected weekly standard Kt/V>2 requires a spKt/V at 0.5 with 6 treatments/week [39]. The spKt/V at 0.6 obtained here per treatment with S^3^ is in total agreement with this requirement [39]. Other short frequent daily dialysis technologies, especially the NxStage System One cycler, also report equal dialysis dose delivery (spKt/V) at 0.6 per treatment [40]. Percent reduction rates of urea, creatinine, and phosphate per dialysis session were in a very close range with 44.3 (38.7–52.9), 46.0 (40.0–54.5) and 44.4 ± 16.7%, respectively. Furthermore, the estimated mass removal of urea, creatinine, and phosphate was 383, 4.6, and 28.3 mmol/session, respectively. Mean phosphate levels were 1.5 mmol/L predialysis and 0.7 mmol/L post-dialysis, and serum calcium levels 2.2 and 2.4 mmol/L, respectively, indicating that both divalent ions essential to prevent bone and vascular disorders remained at optimal targets. On the other side, serum potassium varied between 4.6 and 3.6 mmol/L, and bicarbonate levels also remained in the optimal range to prevent electrolytic disorders and cardiac arrhythmias. Anemia correction and nutritional status tend to be well preserved in this daily treatment schedule, as indicated by mean values of hemoglobin and serum albumin. 

The S^3^ device has unique features that make it almost unique in this therapy field area. S^3^ relies on a bagged distributing monitor system designed in a compact and appealing aspect, easily handled, and truly portable, benefiting from wireless connectivity with a tactile tablet. S^3^ requires only a power supply and waste drainage connection to be activated. Although the use of dialysate in bags may represent a disadvantage in terms of volume (not very different from peritoneal dialysis), it brings many advantages, including the safety of sterile dialysate, the portability of the device, without need to install a water treatment at patient’s home or to provide technical maintenance for dialysate production.

It benefits from a friendly and self-intuitive user interface supported by a tactile tablet offering both constant monitoring and driving of operating dialysis conditions but also a link to the caregiver team facilitating patient support. Dialysis treatment efficacy relies on the daily use of 20–30 L of dialysis fluid adjustable to metabolic patient needs to be applied either in a 2 to 3 h daily five to seven times/week. This flexibility in treatment prescription may help to cover a wide range of dialysis patients’ needs.

This study deserves some limitations. The retrospective and cross-sectional nature of this study did not permit clinical and biological performances regarding the removal of middle molecules in order to validate the additional value of the push-pull technology or patient outcomes by comparison with other daily home dialysis therapies.

## 5. Conclusions

In this report, the safety and efficacy of the S^3^ therapy concept relying on a short daily hemodialysis treatment using a bagged delivery system are in agreement with daily HHD recommendations. Clinical performances are aligned to the metabolic needs of the vast majority of HHD patients. The safety of S^3^ has been confirmed by device clinical performances over a 48-month period of observation corresponding to the duration of the period of inclusion and of the training as a proof-of-concept study of included patients. The compacity and friendly design of S^3^ make it quite appealing for dialysis patients wishing to keep their freedom and mobility opportunities. S^3^ appears to be particularly well suited for HHD therapy, as suggested by our preliminary results in terms of clinical performance and patient acceptance. However, this statement needs to be substantiated by further ongoing HHD studies.

## Figures and Tables

**Table 1:** Characteristics of the study population at baseline. Values were described by using proportions for categorical variables and mean values ± standard deviation or median (percentile 25–percentile 75) for quantitative variables according to their distribution in the total population. (Pre, Predialysis; Post, Postdialysis).

Variables	Total	Male	Female
Demographic variables			
N patients (%)	80 (100.0%)	57 (71.3%)	23 (28.8%)
Incident	12 (15.0%)	9 (15.8%)	3 (13.0%)
Prevalent	68 (85.0%)	48 (84.2%)	20 (87.0%)
Age (years)	54.0 (43.0–63.8)	60.0 (44.0–66.5)	47.0 (38.0–53.0)
Height (cm)	170 (165–176)	172.5 (168.0–178.3)	162.5 (156.8–165.8)
Residual Diuresis (≥0.5 L/d)	47 (58.8%)	34 (59.6%)	13 (56.5%)
Incident	12 (100.0%)	9 (100.0%)	3 (100.0%)
Prevalent	35 (51.5%)	25 (52.1%)	10 (50.0%)
Treatment prescription and clinical parameters			
N sessions per week	6 (5–6)	6 (5–6)	6 (5–6)
Treatment time (min)	120 (120–146)	120 (120–150)	120 (120–120) ^1^
Body Weight Pre (kg)	76.5 ± 17.6	81.3 ± 16.2	64.8 ± 15.4
Body Weight Post (kg)	75.2 ± 17.5	79.9 ± 16.2	63.6 ± 15.2
Body Mass Index (kg/m^2^)	24.9 (21.9–29.3)	25.0 (22.429.7)	24.1 (20.5–28.3)
Qb (mL/min)	280 (280–300)	280 (280–300)	280 (280–300)
Qd (mL/min)	180 (180–180)	180 (180–180)	180 (180–180)
Convective Volume (L)	3.0 (2.0–3.0)	3.0 (2.0–3.0)	2.2 (2.0–3.0)

^1^ All female patients dialyzed almost exactly to prescription; mean values ± SD: 122.7 ± 16.8 min.

**Table 2:** Pre/post-dialysis body weight and ultrafiltration using the Physidia S^3^ daily home dialysis monitor. Values were described by using mean values ± standard deviation or median (percentile 25–percentile 75) according to their distribution.

Parameter	Mean Value ± SD/Median (25–75)
N session analyzed	249
Body Weight predialysis (kg)	79.8 ± 19.4
Body Weight postdialysis (kg)	78.4 ± 19.3
Total ultrafiltration volume (L)	1.3 ± 0.8
Ultrafiltration rate (mL/h/kg)	8.5 (4.0–12.1)

**Table 3:** Biological parameters (uremic control, nutrition, and anemia markers) with use of the Physidia S^3^ daily home dialysis monitor. Values were described by using mean values ± standard deviation or median (percentile 25–percentile 75) according to their distribution.

Parameter	Mean Value ± SD/Median (25–75)	
	*Pre-dialysis*	*Post-dialysis*
Urea (mmol/L)	19.3 (15.3–24.9)	10.2 (7.6–14.0)
Creatinine (µmol/L)	717.5 (547.5–930.0)	370.0 (265.5–493.0)
Potassium (mmol/L)	4.6 (4.1–5.1)	3.6 (3.2–3.9)
Calcium (mmol/L)	2.2 (2.0–2.3)	2.4 (2.3–2.6)
Phosphate (mmol/L)	1.5 (1.2–1.9)	0.7 (0.6–0.9)
Serum Albumin (g/L)	39.4 ± 5.7	-
Hemoglobin (g/dL)	11.6 ± 1.4	-
Hematocrit (%)	35.8 ± 4.5	-

**Table 4:** Clinical performances of the Physidia S^3^ daily home dialysis monitor. (PR, percent reduction; TAC, time average concentration; sp, single pool; eq, equilibrated; WkSd, weekly standardized; nPCR, normalized protein catabolic rate). Values were described by using mean values ± standard deviation or median (percentile 25–percentile 75) according to their distribution.

Parameter	Mean Value ± SD/Median (25–75)
N session analyzed	249
PR Urea (%)	44.3 (38.7–52.9)
Urea TAC (mmol/L)	14.2 (11.4–18.7)
spKt/V	0.66 (0.55–0.83)
eqKt/V	0.59 (0.49–0.75)
WkSdKt/V ^1^	2.22 (1.95–2.61)
Urea Mass (mmol/session)	383 (283–515)
nPCR (g/kg/24 h)	0.93 (0.73–1.18)
PR Creatinine (%)	46.0 (40.0–54.5)
Creatinine Mass (mmol/session)	4.6 (3.4–6.5)
PR Phosphate (%)	44.4 ± 16.7
Phosphate Mass (mmol/session)	28.3 ± 13.7

^1^ Including residual kidney function.

## Data Availability

The data presented in this study are available on request from the corresponding author. The data are not publicly available due to ethical concerns.

## Appendix A

*Calculations*

### Appendix A.1. Dialysis Dose

■ Single Pool Urea Kt/V (spKt/V) was calculated as follows:
  spKt/V = −Ln([urea]_post_/[urea]_pre_) − 0.008 × THD + ((4 − 3.5 × [urea]_post_/[urea]_pre_)(Weight_pre_ − Weight_post_)/Weight_post_)) (A1)
  where [urea] is urea concentration in mmol/L, *pre* and *post* correspond to pre- and post-treatment conditions. respectively. THD is time on dialysis in hours and Weight is body weight in kg.
■ Equilibrated Kt/V (eqKt/V) was calculated as follows:
  eqKt/V = spKt/V − (0.6 × spKt/V/THD) + 0.03 (A2)
  where THD is time on dialysis in hours.
■ Weekly standardized Kt/V (WkSdKt/V) was calculated as follows:
  WkSdKt/V = [10.080(1 − e^−eqKt/V^)/t]/[((1 − e^−eqKt/V^)/eqKt/V) + (10.080/Ft) − 1] (A3)

### Appendix A.2. Solute Mass Removal

Solute mass removal for solutes of interest (urea, creatinine, and phosphates) was calculated as follows:

Mass solute = K solute × TAC solute × THD (A4)

where K solute is solute clearance and equals to:

K solute = Ln(C_pre_/C_post_) × TBW/THD (A5)

C is solute concentration; *pre* and *post* correspond to pre- and post-treatment conditions, respectively. TBW is total body water estimate from empirical postdialysis weight as 0.55 for male and 0.50 for female. THD is time on dialysis. TAC solute is time average concentration of the solute and equals to

TAC solute = (C_pre_ − C_post_)/Ln(C_pre_/C_post_) (A6)

### Appendix A.3. Normalized Protein Catabolic Rate (nPCR)

nPCR derived from urea apparition rate was calculated as follows:

nPCR = (0.0504 × (1 − 0.162 × ([urea]_post_/[urea]_pre_)) × (1 − ([urea]_post_/[urea]_pre_) + ((Weight_pre_ − Weight_post_)/(Weight_post_ × 0.58 (if male) or × 0.53 (if female))))) × ([urea]_pre_/(1 − (0.0003 × (THD/60)))) + 0.17 (A7)

Of note, the calculation of the nPCR index depends upon including the intrinsic renal function of the patients. Since urine samples were not collected in this study, the nPCR index calculated here is based on the assumption that all patients were anuric and, therefore, tends to be underestimated.
